# A Solar Energy Powered Autonomous Wireless Actuator Node for Irrigation Systems

**DOI:** 10.3390/s110100329

**Published:** 2010-12-30

**Authors:** Rafael Lajara, Jorge Alberola, José Pelegrí-Sebastiá

**Affiliations:** Research Institute for Integrated Management of Coastal Areas, Universitat Politècnica Valencia, EPSG, C.Paranimf, 1, Gandía, Spain; E-Mails: jolaviz@doctor.upv.es (R.L.); jalberolalluch@gmail.com (J.A.)

**Keywords:** wireless sensor networks, actuator node, autonomous sensor, solar energy, agriculture irrigation systems

## Abstract

The design of a fully autonomous and wireless actuator node (“wEcoValve mote”) based on the IEEE 802.15.4 standard is presented. The system allows remote control (open/close) of a 3-lead magnetic latch solenoid, commonly used in drip irrigation systems in applications such as agricultural areas, greenhouses, gardens, *etc.* The very low power consumption of the system in conjunction with the low power consumption of the valve, only when switching positions, allows the system to be solar powered, thus eliminating the need of wires and facilitating its deployment. By using supercapacitors recharged from a specifically designed solar power module, the need to replace batteries is also eliminated and the system is completely autonomous and maintenance free. The “wEcoValve mote” firmware is based on a synchronous protocol that allows a bidirectional communication with a latency optimized for real-time work, with a synchronization time between nodes of 4 s, thus achieving a power consumption average of 2.9 mW.

## 1. Introduction

Localized irrigation systems require the use of latch type solenoids to control automated irrigation zones. Currently, control is achieved by using wired networks with two or three leads to communicate and carry out the actions (activation/deactivation) of the solenoid, such as the Piccolo RTU from Motorola [1]. Some newer systems are based on a 2.4 GHz radio link [2–5] but do not implement the control of the solenoid valve. Most of them, normally, are used for monitoring environmental conditions. There are other systems that take control when implemented, such as Piccolo-XR [1] but they need a battery, which must be replaced from time to time in order to assure the action of the solenoid.

Wireless sensor networks have the peculiarities that, as a rule, do not use large data transfer rates, and furthermore, their nodes are powered by small batteries and/or supercapacitors. Our design consists of a hardware part, where the components were chosen to optimize the use of the available energy. The firmware has also been taken into account for this purpose, as the main goal of a MAC layer protocol for these devices is to save energy. This requires to identify the main mechanisms of energy cost as the detailed in [6], hence the importance of timing in this type of nodes and applications.

Traditional MAC protocols keep the receivers always listening to a channel, since energy efficiency is not priority [7], but this is not practical in low power sensor networks, where energy efficiency is a major concern. Thus the sensor network nodes should power-off their transceivers when they have a chance [8]. These reasons and others such as hardware limitations or the need for reconfiguration make traditional synchronization mechanisms not valid in low power sensor networks, and new approaches claim to be developed specifically, even for each type of application in particular [7–13].

For two sensor nodes to communicate with each other, they must enable their transceivers within the same period of time (synchronously), one in transmission mode and the other in reception mode. The problem to be solved by the MAC protocol is to determine when to enable the transceiver, having into account that there must be synchronization between at least two of the nodes. In addition they must also take into account the time when two nodes want to transmit at a time to avoid collisions, and when a node wants to transmit data but must also receive.

The main energy saving is achieved by disabling the transceiver when not needed. For example, the CC2420 transceiver, used in this node consumes 19.7 mA in transmission, 17.4 mA in reception and 20 μA in power-down mode [14]. Of course, the microprocessor must be waked up from its idle or sleep mode before enabling the transceiver. In addition it would also be desirable for other devices such as sensors and/or actuators be disabled when not needed.

Activating devices only when they are needed, leads to the typical listen/sleep duty cycles of low power sensor networks applications. In active mode, the node will perform tasks as transmitting or sensing, and in sleep mode it will turn off unused devices. For simplicity consider that the processor and the sensors have the same activation period of the transceiver. The lower is the duty cycle, the lower is the average consumption.

## 2. wEcoValve Hardware Development

**Figure 1.**
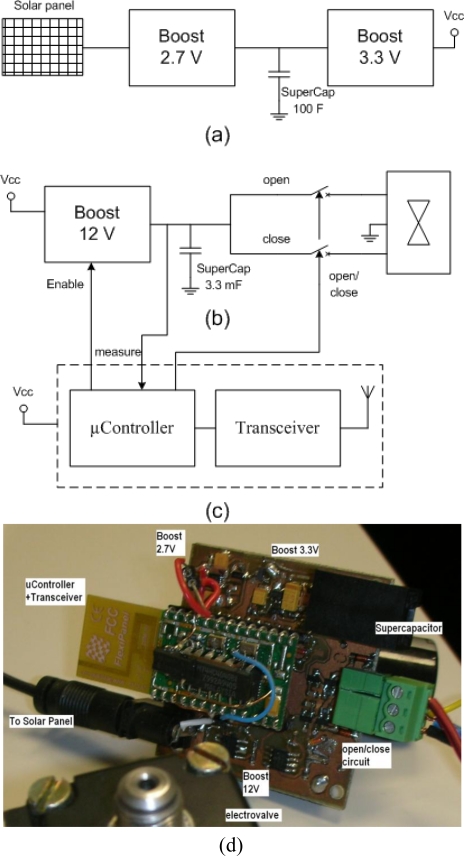
Block diagram of a terminal node. **(a)** Power circuit formed by the solar panel, a supercapacitor and two highly efficient DC-DC converters; **(b)** Control circuit of the electrovalve formed by a DC-DC converter, a supercapacitor and a discharge circuit to control the electrovalve; **(c)** Microcontroller and tranceiver block diagram with a Flexipanel Pixie module; **(d)** A photo of the wEcoValve laboratory prototype.

The “wEcoValve mote” block diagram is shown in Figure 1, where there are three sections: (a) the supply circuit formed by the solar panel, a supercapacitor and two highly efficient DC-DC converters to supply a regulated 3.3 V to the microcontroller and the transceiver; (b) the control circuit for the solenoid, which comprises a DC-DC converter, a supercapacitor and a circuit to control the solenoid valve with a 12 V supply; (c) diagram of the microcontroller and the transceiver, implemented by a Flexipanel Pixie module.

The actuator node is powered by a circuit similar to the one shown in [15], but adapted to the needs of the action over the solenoid valve and based on the use of a solar panel that recharges a single 100 F supercapacitor to be fully autonomous without replaceable batteries.

The Epson SG-3030JC oscillator and the 74HC40103 counter have been used to divide the clock frequency down and allow the microcontroller to work in the lowest power mode, as explained later. Current pulses of 700 mA and 12 V peak and 20 ms long open and close the solenoid valve [16]. These pulses are achieved through a 12 V Boost Converter and a second 3.3 mF supercapacitor that stores energy for activation/deactivation of the solenoid. The supercapacitor is charged up to 10.5 V, and this voltage is measured by the microcontroller by means of polling. Once the supercapacitor has accumulated enough energy, the triggering circuit of the solenoid valve activates the discharge of the supercapacitor with a 10 μs voltage pulse. Finally, other circuits were added to measure the current provided by the solar panel and to measure the voltage across the main supercapacitor for testing and debugging purposes.

## 3. Synchronization Protocol Development

In [7] a classification of different types of protocols was given, highlighting the slotted (TDMA, 802.15.4, SMAC, DSMAC, TMAC, FRAME), the sampling (ALOHA, BMAC, WiseMAC, CSMA; (for wireless networks using CSMA/CA)) and the others (STEM (using two transceivers, one for data transfer with a certain listen/sleep duty cycle, and the other one that is permanently activated to wake-up the system) and hybrids (e.g., ZMAC, SCP-MAC)).

Slotted protocols keep nodes permanently synchronized, so they are particularly well suited for applications where there is a periodic exchange of information. Also they know exactly when to be activated, allowing them to shorten the active periods. Moreover, sampling protocols will be more convenient in terms of energy in applications with sporadic transmissions, because there is no penalty to maintain the synchronization.

### 3.1. Slotted Protocols

Slotted protocols require that all nodes work with a common clock signal, which will serve to maintain synchronization. In the end, at the lowest level, all of these protocols use the main crystal of the system to maintain the synchronization. If these clocks were ideal, after initialization, the nodes would always be perfectly synchronized, however frequency variations occur between clocks from one node to another due to various causes, such as temperature changes, aging or frequency stability [13].

For example, the clock used in the Microchip PICDEM.Z evaluation board, the HC-49US from ECS International Inc., has a frequency stability of ±15 ppm to ±100 ppm at 25 °C [17]. This means that at worst, within 1 minute the clocks of two nodes have been lagged 12 ms. To maintain synchronicity one can send special frames (beacons) broadcasted periodically or use the preamble of the messages to send information about the synchronization. In both cases, drift margins have to be taken into account to avoid possible desynchronizations.

MAC protocols focus on activating and deactivating the transceiver, and this requires the microprocessor to maintain the synchronization. This entails a problem if you want to set the microprocessor to a low-power mode: the microprocessor maintains the synchronism, but if all of the devices (including timers) were turned off, the synchronization would get lost.

The PIC18F4620 microcontroller used has a clock frequency of 4 MHz and 3 V draws 1.3 mA in active mode, 430 μA in PRI_IDLE mode (CPU and peripherals switched off) and 0.1 μA deep sleep mode [17]. For a duty cycle of 1% and a 21 mA current consumption in listening mode (microcontroller plus transceiver active), and a current consumption of 450 μA in sleep mode (PRI_IDLE mode microcontroller and transceiver powered down), the overall average consumption is 655.5 μA.

However the use of microcontroller’s deep sleep mode (0.1 μA) can achieve an average consumption of 210.1 μA. For that, the aim is to change the microcontroller from one mode to another during its sleep period, thus reducing the consumption by two thirds.

### 3.2. The Proposal

Slotted protocols with a low duty cycle will approach the average consumption to the sleep mode consumption. Therefore, reducing this consumption has a major impact on the average consumption. The aim is to optimize the power consumption for a given protocol by reducing the current drawn in sleep mode. To do this, one needs to bring the microprocessor to the lowest power mode and stay there as much time as possible. For that, all the peripherals and even the processor must be powered down, all but some system to cause wakeup again.

The first approach would be to use some internal oscillator to periodically wake the processor up. For example, the PIC18F4620 microcontroller incorporates an internal 32 kHz oscillator [18] connected to the watchdog timer, which can be used for this task. But this presents two downsides: 32 kHz is too high a frequency for low duty cycle applications of low power sensor networks, so the processor probably wakes up more often than necessary. This can be solved by using the prescaler and postscaler counters of the watchdog circuit. The second and main problem is that this oscillator is an RC type, which means that it has worse stability than those based on quartz crystals. The microcontroller under study can vary its frequency from 26.562 kHz to 35.938 kHz, which makes a count that may be 1 s long differs up to 0.3 s at the worst case. This problem makes to discard the use of RC oscillators. It is also the reason why so far the quartz crystal and a counter have been used as the main oscillators and as the basis of synchronism.

**Figure 2.**
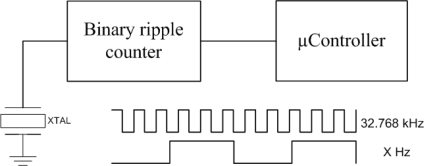
Introduction to a binary ripple counter that permits the microcontroller to enter sleep mode.

Instead of using the internal RC oscillator, a simple circuit consisting of a low frequency crystal oscillator and a frequency divider has been added, see Figure 2. The output of this circuit is connected to an input that triggers an interrupt, which wakes up the processor. This solution allows turning all the peripherals of the processor fully off, so that the consumption in sleep mode is considerably reduced.

However, when using an external oscillator of 32.768 kHz, consumptions and tolerances can be widely improved. The oscillator that we take as an example is the 90SMX from IQD [19], which has a maximum frequency tolerance of ±100 ppm (typically ±20 ppm). The worst case is when two nodes are desynchronized the same time as the preceding example, where there was a 4 MHz clock with a 100 ppm tolerance, *i.e.*, 12 ms over 1 minute.

This accuracy is more than enough for typical applications in low power sensor networks such as drip irrigation. In fact sometimes the processor’s wakeup time may be more relevant than the imprecision of the clocking. With this solution, there is sufficient accuracy for the time measurement. Concerning the consumption, the crystal has its maximum value of 1 μW. On the other hand the PIC18F4620 microcontroller can be brought to its lowest power consumption mode, so-called sleep (OSCON = 0), whose consumption is 0.3 μW (0.1 µA at 3 V).

By adding a frequency divider like the 74HC40103 for the oscillator, a maximum current of 8 μA at 6 V must be added. The use of this divider allows the microcontroller to enter the sleep mode most of the time, saving energy (see Figure 2). Therefore there is a power consumption of 1 μW (oscillator) + 0.3 μW (microcontroller in sleep) + 12 μW (frequency divider at 3 V). That is a total of 13.3 μW.

A typical synchronization scheme would save the low frequency external clock and the frequency divider, but the processor could not enter the deepest sleep mode. The previous microcontroller would have to use an internal counter to be active, therefore it would have to enter the PRI_IDLE mode. This entails a consumption of 430 μA at 3 V and 4 MHz for the CPU and 2.6 μA for the internal counter of the microcontroller. It would be 1.3 mW in total.

The power consumption of the transceiver in sleep mode and the rest of the device should be added in both measures, but the consumption is reduced by 100 times only considering the components that affect the synchronization.

## 4. “wEcoValve” Firmware/Software Development

**Figure 3.**
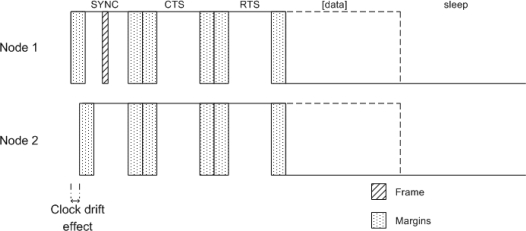
Scheme listen/sleep from two nodes. The second node starts its listen period after the listen period of the first node. When node 1 broadcasts the SYNC frame, both nodes get resynchronized. The nodes reserve margins (dotted area) in each sub-period (SYNC, CTS and RTS) of listen mode in order to avoid the loss of a frame when other nodes are still not listening. The frames are sent passed a random time, after the beginning of each sub-period, respecting the safe margins. When a node receives a frame in a sub-period, it must await for the next listen period.

The solution proposed in Section 3.2 has been implemented together with the SMAC protocol [20]. This protocol allows two or more nodes to synchronize and communicate with each other during their active period. The synchronization protocol is accomplished with regular broadcast transmission of SYNC frames from nodes to nodes; the nodes update their timers to keep synchronized by taking the time from these frames. A scheme based on containment through frames CTS/RTS is used to avoid collisions. RTS frames are a request to send data and CTS is the confirmation. Data frames are sent after that if needed. When there is no data to broadcast, the node enters sleep state. A scheme of these frames can be seen in Figure 3.

**Figure. 4.**
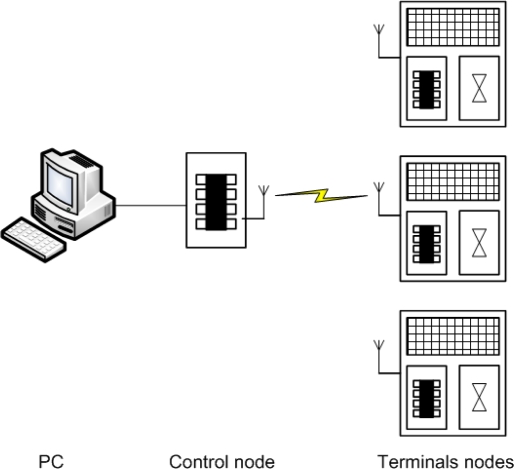
Scheme of communications between the “control nodes” and “terminals nodes”.

Some of the nodes that form the network are called “control nodes”, and transmit the open and close commands to remotely control the solenoid valves. Other nodes are called “terminal nodes”, and are the ones that receive the commands for actuating over the solenoid valves. The latter shall be responsible for monitoring the valve states (cf. Figure 4). Furthermore, to assist in debugging the application, “terminal nodes” broadcast the current value supplied by the solar panel and the voltage value across the supercapacitors. This information also aids to know the status of each node at any moment. Thus it could be used to know whether a node might fail for not being able to open/close the solenoid, or might not become active again due to the lack of energy. This information is received by the “control node”, and passed through a serial communication to an application running on a PC. This application allows the user to broadcast opening or closing commands from a “control node” to each valve.

## 5. Results and Discussion

**Figure 5.**
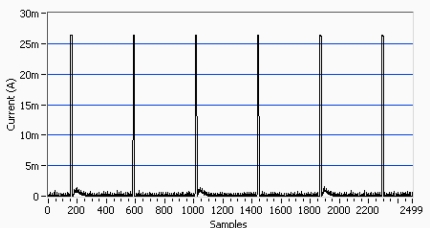
Current consumption of a terminal node. The peaks of 27 mA are produced every 4 seconds due to the synchronization between nodes. The average consumption is 836.76 μA.

The remotely controlled “wEcoValve” has been implemented by using the SMAC protocol. In the implementation a 4 seconds period of resynchronization has been chosen, see Figure 5. Every 4 seconds a node broadcasts a synchronization frame to be listened by all its neighbors. Since there is a random delay before a node broadcasts a SYNC frame, the node with the shortest delay updates the time of the rest. Thus, the node which transmits the SYNC frame remains uncertain, and it is randomly selected.

The resynchronization time can be increased, whereas the clock’s drift, not evident. In the same manner this time also can be reduced to improve the latency. A monitoring application has also been implemented on the PC, which can send data via RS232 to a “control node” in order to open and close the “terminal nodes”. This can be scheduled.

**Figure 6.**
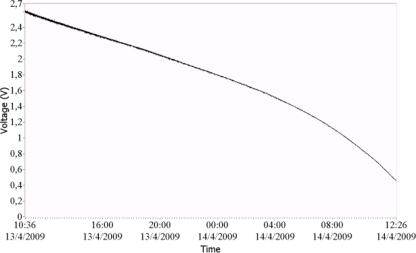
Supply supercapacitor discharge curve of the wEcoValve mote. It has a lifetime of approximately 26 hours without solar energy support. The synchronization period is set to 4 seconds. A lifetime of 50 hours can be reached with a synchronization period of 8 seconds.

**Figure 7.**
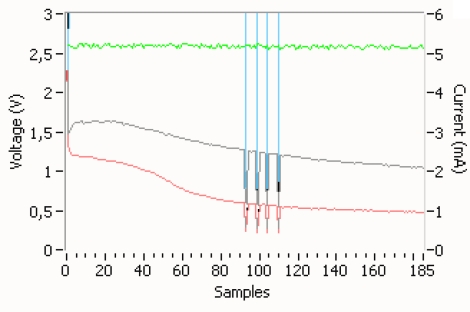
Supercapacitor discharge (green) for two consecutive opening and closing actions of the solenoid (in blue).

Experimental measurement results of the current generated by the solar panel and the voltage by the supercapacitor are shown in Figures 6 and 7. In Figure 6 the supply supercapacitor discharge curve of the wEcoValve mote has a lifetime of approximately 26 hours without solar energy support, with a synchronization period of 4 seconds. A lifetime of 50 hours can be reached with a synchronization period of 8 seconds. Figure 7 presents the supercapacitor discharge curve (green) for two consecutive opening and closing actions of the solenoid (in blue).

**Figure 8.**
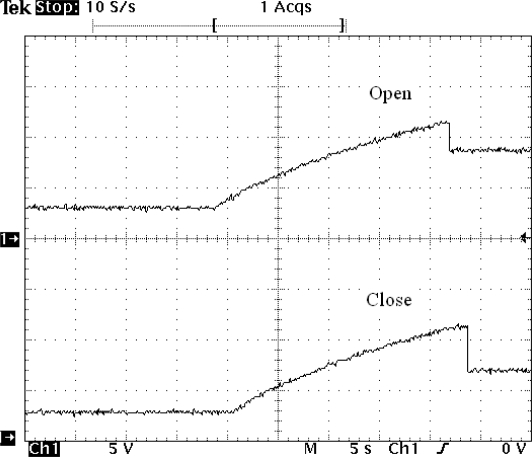
**(a)** Open. A short pulse (microseconds) initiates the charge of the boost’s capacitors up to the opening/closing threshold. Once the threshold is reached (about 11.4 V), a 15 ms pulse is long enough to open the valve, and this action drops about 3 V. **(b)** Close. A 20 ms pulse is long enough to close the valve, with a voltage drop of about 5 V.

On the other hand the hardware developed (Figure 1(b)) to open/close the solenoid has been optimized so that the charging and discharging of the supercapacitor of 3.3 mF only occurs when requested and by using as little energy as possible. This can be seen in Figure 8, where a short pulse (microseconds) initiates the charge of the boost’s capacitors up to the opening/closing threshold.

**Figure 9.**
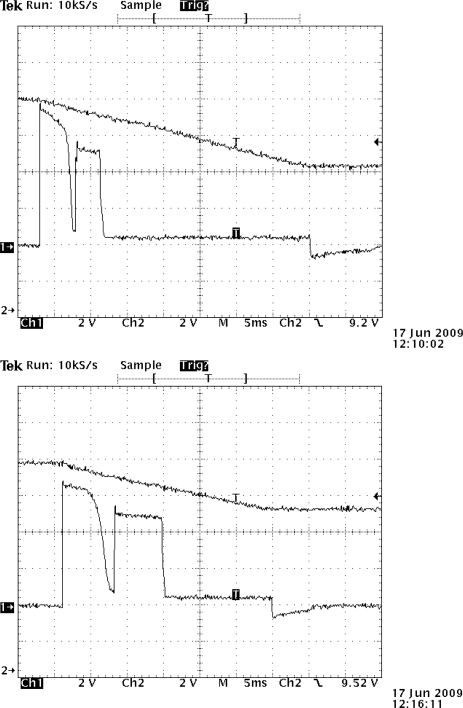
Ch1 is the current that triggers the solenoid and ch2 is the voltage of the capacitor of 3.3 mF.

Once the threshold is reached (about 11.4 V), a 15 ms pulse is long enough to open the valve, and this action drops about 3 V (Figure 8(a) open). Figure 8(b) is closed; a 20 ms pulse is long enough to close the valve, with a voltage drop of about 5 V. Figure 9 shows the current necessary for the activation and deactivation of the solenoid.

Neither the opening nor the closing actions fully discharge the boost’s capacitor (3.3 mF), leaving some residual charge which might vary from 7 V to 9 V, thus allowing for quicker consecutive loads with delays of only 3 s in the worst case, unlike a complete charging time that takes 23.1 seconds for both opening and closing the solenoid valve. The voltage threshold that allows fully triggering the valve is approximately 11.4 V and the voltage drop caused by the switching normally is 2.8 V when opening and 4.6 V when closing.

## 6. Conclusions

A fully autonomous and remote solenoid valve node (wEcoValve) that can be used for irrigation of gardens, indoor plants, greenhouses or field crops has been implemented and tested. It is in general suitable for any system that requires a solenoid type latch valve.

Its key advantage is that it does not need any cabling deployment to power the node and the electrovalve, because it is wirelessly controlled and solar powered. This was accomplished with an ultra low power consumption hardware/firmware design that allowed supercapacitors (100 F) to provide the system’s power supply for more than a day without direct sunlight over the solar panel, depending on the synchronization period and the duty cycle of use in the end application. The use of supercaps with almost no degradation over time eliminates the need for batteries replacement.

A very precise synchronization method (4 seconds for resynchronization) has been designed by making use of a low frequency crystal and a frequency divider, which let us to shut down most of peripherals, thus reducing considerably the power consumption. It can be used in several synchronization protocols to reduce messaging traffic or improve accuracy. It could be used to open or close electrovalves that control the water flow through plants. Each solenoid could be connected to a node that could be wirelessly and independently controlled.

As future work, we have started to test the board in a real scenario (orange fields in south Valencia) and collect data. Later the system could be adapted for measuring the water flow and to keep track of the consumed water, plus transmitting the data out to a central station.

## References

[1] (2009). Irrinet for a Freener World. Irrinet System Description. Product Brief.

[2] Lopez J.A., Soto F., Sanchez P., Iborra A., Suardiaz J., Vera J.A. (2009). Development of a sensor node for precision horticulture. Sensors.

[3] (2009). eKo Outdoor Wireless System for Environmental Monitoring.

[4] Kim Y, Evans R.G., Iversen W.M. (2008). Remote sensing and control of an irrigation system using a distributed wireless sensor network. IEEE Trans. Instrum. Meas.

[5] Chavez J.L., Pierce F.J., Eliott T.V. (2010). A remote irrigation monitoring and control system for continuous move systems. Part A: Description and development. Precis. Agric.

[6] Demirkol I., Ersoy C., Alagoz F. (2006). MAC protocols for wireless sensor networks: A survey. IEEE Commun. Mag.

[7] Kuorilehto M., Kohvakka M., Suhonen J., Hämäläinen P., Hännikäinen M., Hamalainen T.D. (2007). Ultra-Low Energy Wireless Sensor Networks in Practice.

[8] Sichitiu M.L., Veerarittiphan C. (2003). Simple, accurate time synchronization for wireless sensor networks. Wirel. Commun. Netw.

[9] Elson J., Römer K. (2003). Wireless sensor networks: A new regime for time synchronization. Sigcomm. Comput. Commun. Rev.

[10] Faizulkhakov Y.R. (2007). Time synchronization methods for wireless sensor networks: A survey. Program. Comput. Soft.

[11] Sommer P., Wattenhofer R. Gradient clock synchronization in wireless sensor networks.

[12] Sundararaman B., Buy U., Kshemkalyani A.D. (2005). Clock synchronization for wireless sensor networks: A survey. Ad Hoc Netw.

[13] (2009). CC2420, 2.4 GHz IEEE 802.15.1/ZigBee-Ready RF Transceiver Data Sheet.

[14] Alberola J., Pelegri J., Lajara R., Perez J.J. Solar inexhaustible power source for wireless sensor node.

[15] Pelegri J., Ramírez D. (1999). Circuit latches solenoid at a distance. EDN Mag.

[16] HC-49US Quartz Crystal Datasheet.

[17] Enahnced Flash Microcontroller with 10-Bit A/D and nanoWatt Technology Data Sheet.

[18] (2009). 90SMX & 91 SMX Crystals Data sheet from Datasheet.

[19] Lajara R., Pelegri-Sebastia J., Perez-Solano J.J. (2010). Power consumption analysis of operating systems for wireless sensor networks. Sensors.

[20] Wei Y., Heidemann J., Estrin D. An energy-efficient MAC protocol for wireless sensor networks.

